# Synergistic Effects of *Trichoderma harzianum*, 1,3 Dichloropropene and Organic Matter in Controlling the Root-Knot Nematode *Meloidogyne incognita* on Tomato

**DOI:** 10.3390/plants11212890

**Published:** 2022-10-28

**Authors:** Giada d’Errico, Nicola Greco, Francesco Vinale, Roberta Marra, Virgilio Stillittano, Salvatore Walter Davino, Sheridan Lois Woo, Trifone D’Addabbo

**Affiliations:** 1 Department of Agricultural Sciences, University of Naples Federico II, 80055 Portici, NA, Italy; 2 Institute for Sustainable Plant Protection—CNR, 70126 Bari, BA, Italy; 3 Department of Veterinary Medicine and Animal Productions, University of Naples Federico II, 80055 Portici, NA, Italy; 4 Experimental Zooprophylactic Institute of Latium and Tuscany “M. Aleandri”, 00178 Roma, RM, Italy; 5 Department of Agricultural, Food and Forest Sciences—University of Palermo, 90133 Palermo, PA, Italy; 6 Department of Pharmacy, University of Naples Federico II, 80055 Portici, NA, Italy

**Keywords:** *Meloidogyne incognita*, integrated management, 1,3 dichloropropene, *Trichoderma harzianum*, organic matter

## Abstract

Environmental concerns raised by synthetic nematicides are encouraging integrated management strategies based on their combination with non-chemical control tools, such as biocontrol agents and/or organic amendments. In this study, the combination of the fumigant 1,3-dichloropropene (1,3-D) with a commercial formulation of the biocontrol agent *Trichoderma harzianum* (TH) and an organic fertilizer (OF) was investigated in two consecutive tomato crops for its effect on the root-knot nematode *Meloidogyne incognita* and plant growth and yield. The application of 1,3-D was only performed on the first crop, while TH and OF were provided to both crops. Almost all treatments significantly reduced nematode infestation in both crops, though the greatest nematicidal effect was caused by a combination of the three products. The treatment with 1,3-D limited its nematicidal efficacy to the first crop only. Fumigant integration with TH and OF also resulted in the greatest increases of plant growth and yield. Therefore, the integrated management of root-knot nematodes with a soil fumigant, a bionematicide as *T. harzianum* and a source of organic matter demonstrated effective nematode suppression though limiting the number of chemical applications.

## 1. Introduction

Phytoparasitic nematodes, and particularly the root-knot species of genus *Meloidogyne*, are the cause of huge yield losses on a large spectrum of horticultural and ornamental crops in all tropical and subtropical regions of the world [1]. Crop losses attributable to phytonematodes annually amounts around 12.3% of the total world agricultural production, corresponding to an economic value of USD 157 billion, versus the USD 70 billion losses caused by invasive insects [2,3]. Moreover, nematode damages are probably underestimated due to the non-specificity of plant symptoms, as well as due to the lower quality of crop yield and possible interactions between nematode and fungal pathogens [4,5,6].

Control of phytoparasitic nematodes has been traditionally based on soil treatments with synthetic nematicides, mainly fumigants, but concerns for their environmental and health effects have led to a complete ban or restrictions on the use of most nematicides while also adhering to the European Community Regulations [7,8]. Among fumigant nematicides, 1,3-dichloropropene (1,3-D) has been repeatedly acknowledged for a high effectiveness against root-knot nematode infestations and positive effects on crop yield [9,10,11]. This product is still widely applied in horticultural areas thanks to renewals of annual derogation of the law for use on a limited numbers of crops during a limited period of the year, usually four months.

The need of nematode control tools as an alternative to chemicals has strongly encouraged research on the use of biocontrol agents, generally approved by public opinion and encouraged by EU policy. Fungi of genus *Trichoderma* are among the most promising biocontrol agents, in addition to mycorrhizal and endophytic fungi [12]. The nematicidal effectiveness of *Trichoderma* spp. has been recently proved by both semi-field studies [13,14] and field trials [15,16,17]. In addition to a direct nematode suppression, *Trichoderma* fungi can enhance plant resistance against nematode attacks through the activation of hormone-mediated defence mechanisms [18], the synthesis of secondary metabolites and enzymes and an altered translocation of plant chemical defence components [19,20,21,22,23]. *Trichoderma* species were also acknowledged as plant biostimulants, due to the larger availability of water and nutrients related to modified root morphology and rhizosphere interactions [24]. The nematicidal efficacy of *Trichoderma* varies among the fungal strains and is strongly affected by soil physicochemical and biological properties. In particular, a key role is played by the soil content of organic matter (OM) that ensures a feeding substrate for *Trichoderma* development also in the absence of host plants [25], demonstrating a direct suppressiveness to phytoparasitic nematodes, which act synergistically to the nematicidal activity of *Trichoderma* [26,27].

Interestingly, literature data indicated the high resistance of *Trichoderma* spp. to soil treatments with fumigants and their rapid recolonization of soil after chemical treatment, mainly due to the biological vacuum caused by fumigation and the consequent absence of *Trichoderma* predators [28,29]. Therefore, as the fungal establishment in agricultural soils is normally slow and related to the occurrence of favourable pedoclimatic conditions, the soil application of *Trichoderma* after the treatment with a fumigant nematicide could ensure a rapid and permanent establishment of the fungus in the soil, thus, enhancing its nematicidal effectiveness. According to this hypothesis, we investigated the nematicidal performance of combined treatments with the fumigant 1,3-D and a formulate of *Trichoderma harzianum* (TH) in two consecutive tomato crops, either with or without the addition of an organic fertilizer (OF) as a source of OM, against the root-knot nematode *Meloidogyne incognita*.

## 2. Results

### 2.1. Effects on Nematode Infestation

**Figure 1:**
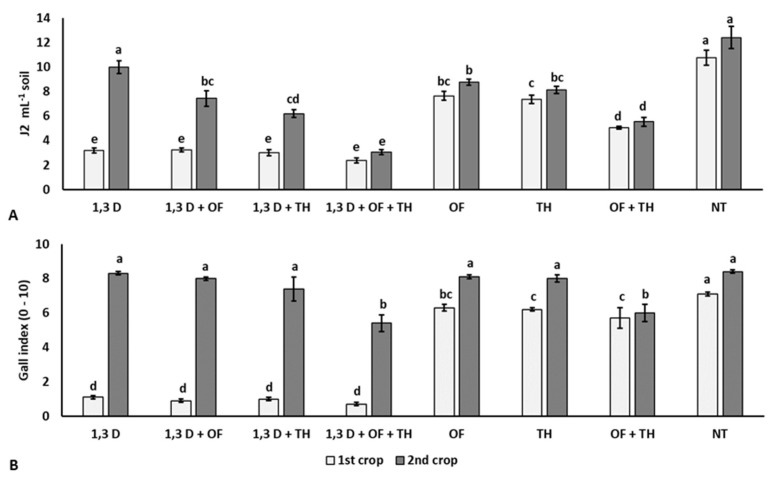
Effects of soil treatments with 1,3-dichloropropene (1,3-D), *Trichoderma harzianum* (TH) and an organic fertilizer (OF), either alone or in combination, on final population density (**A**) and root gall infestation (**B**) of *Meloidogyne incognita* in two consecutive tomato crops. NT = non-treated control. Bars show the average of *n* = 4 replicates of each treatment ± standard error. Within each tomato crop, bars marked by the same letters are not significantly different, according to the least significant difference test (*p* ≤ 0.05).

At the beginning of the experiment, the soil population density of *M. incognita* ranged from 2.1 to 2.6 *J2* mL^−1^ soil, without statistical difference among the experimental plots (data not shown). At the end of the first tomato crop, all the treatments significantly reduced soil nematode population and root gall infestation index, compared to the non-treated control (NT) (Figure 1). Suppression of *M. incognita* infestation was greater in soil treated with 1,3-D, both alone or combined with TH and/or OF, than in plots treated with TH or OF alone or in their combination and the non-treated control. The combination of fumigation with both non-chemical products resulted in the lowest nematode population. Additionally, the combined treatment of OF and TH resulted in significantly lower numbers of *M. incognita J2*, compared to their single application and the non-treated control. At the end of the second tomato crop, the nematode population in the treated plots was also significantly lower than in non-treated soil, except that in the soil only treated with 1,3-D in the first cycle. As in the first tomato crop, the lowest number of *M. incognita J2* and root gall infestation indices occurred for the coupled application of OF and TH, mainly when previously treated with 1,3-D.

### 2.2. Effects on Tomato Yield

**Figure 2:**
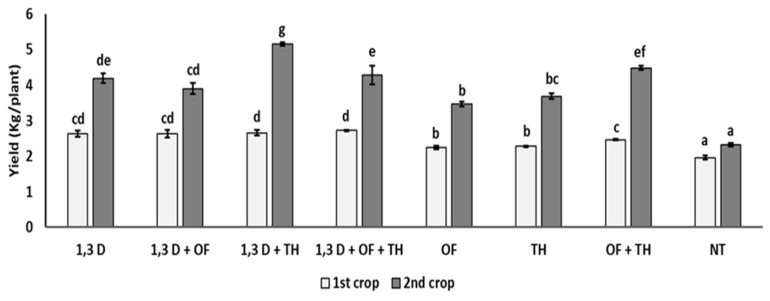
Effects of soil treatments with 1,3-dichloropropene (1,3-D), *Trichoderma harzianum* (TH) and an organic fertilizer (OF), either alone or in combination, on yield (kg/plant) of two consecutive tomato crops. NT = non-treated control. Bars show the average of *n* = 4 replicates per treatment ± S.E. Within each tomato crop, bars marked by the same letters are not significantly different, according to the least significant difference test (*p* ≤ 0.05).

Yield of the first tomato crop was significantly increased by all the tested treatments, though the yield increase provided by OF and TH was statistically greater only when used in combination or with 1,3-D (Figure 2).

In the second tomato crop, tomato yield was always almost double that in the first cycle. As in the first cycle, all the treatments provided a significantly higher yield performance, compared to the non-treated control, though the highest yield values were recorded for the combined application of 1,3-D and TH and, to a lesser extent, of OF and TH.

### 2.3. Effects on Tomato Plant Growth

**Figure 3:**
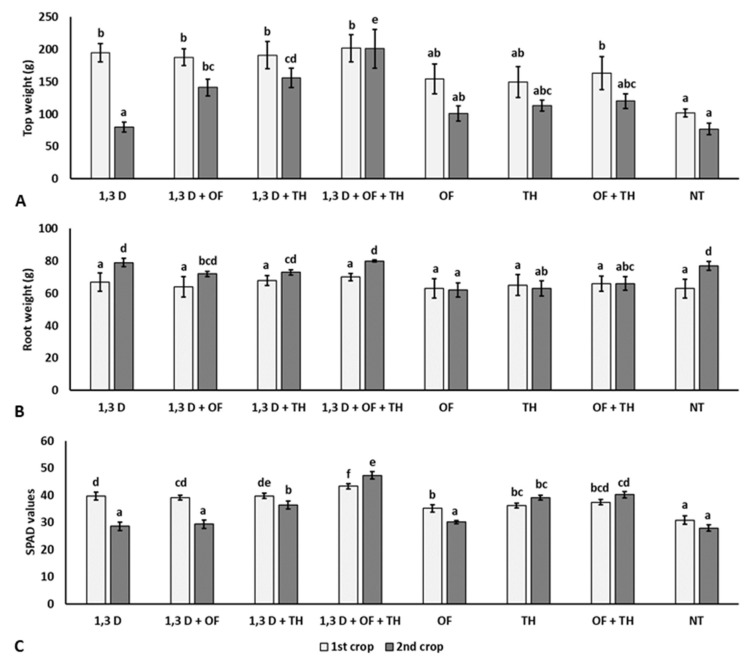
Effects of soil treatments with 1,3-dichloropropene (1,3-D), *Trichoderma harzianum* (TH) and an organic fertilizer (OF), either alone or in combination, on weight of aerial parts (**A**), roots (**B**) and chlorophyll content (**C**) of plants from the two tomato crops. NT = non-treated control. Bars show the average of *n* = 4 replicates of each treatment ± S.E. Within each tomato crop, bars marked by the same letters are not significantly different according to the least significant difference test (*p* ≤ 0.05).

Compared to NT, all the treatments applied in the first crop resulted in a significantly heavier plant biomass and chlorophyll content, though not affecting the root system (Figure 3).

Conversely, the growth of plants from the second crop was only significantly increased in the plots previously treated with 1,3-D in combination with OF and/or TH. Significantly larger root systems and higher chlorophyll contents were also recorded for the combined application of TH and OF.

## 3. Discussion

The RKN *M. incognita* was able to reproduce on tomato Shiren F1 in both crop cycles, in spite of the generally stated resistance of this hybrid to RKN. This can be explained by the virulence of the nematode population present in the greenhouse and its ability to break the tomato resistance, as confirmed by the susceptibility of Shiren F1 to RKN observed in the previous tomato crops of the surrounding areas. A higher nematode reproduction and gall formation on tomato roots was observed in the second crop cycle, as a consequence of the higher soil temperatures occurring in spring–summer conditions.

The increasing attention to environmental safety and human health has led to a dramatic reduction in synthetic pesticides, in favour of more environmentally friendly alternatives, such as the integrated control strategy positively tested in this study. Data presented here indicate that the combined use of soil fumigation with a biocontrol agent, such as *T. harzianum*, and an organic fertilizer can be an effective strategy for root-knot nematode management by enhancing nematicidal effect and yield performance of each of its components and extending the nematicidal effect of the fumigant to a second crop cycle. Our findings agree with recent studies [30,31] documenting a number of favourable effects of soil addition with biocontrol agents (*T. harzianum*, *B. subtilis*) and OFs following 1,3-D fumigation, such as significant reductions of soilborne phytopathogens (*Fusarium* spp., *Phytophthora* spp.), positive changes of soil physicochemical properties, a larger abundance of beneficial microorganisms and an increased crop yield and quality.

Soil fumigation with 1,3-D has demonstrated to be highly effective for controlling root-knot nematode infestation and increasing yield in the first tomato crop, as also previously documented by many other studies either on tomato or other horticultural crops [9,10,11]. In this study, the positive performance of single fumigation with 1,3-D on nematode infestation and plant growth was limited only to the first tomato crop, thus, confirming the short-time nematicidal effects of soil fumigants in medium to highly RKN-infested soil. In the first tomato crop, the nematicidal effect of 1,3-D was not affected by the combination with TH or OF but significantly improved when combined with both products. Moreover, both non-chemical treatments significantly improved the residual nematicidal effect of 1,3-D in the second cycle, mainly when applied in combination. The enhanced effects of soil treatments with Trichoderma products following soil fumigation can be related to the quick recolonisation of fumigated soil by Trichoderma species in the biological vacuum created by fumigation and particularly in the absence of fungal predators [28,29].

The genus Trichoderma includes several species of free-living soil-borne fungi commonly present in roots, often reported as effective against phytoparasitic nematodes and phytopathogenic fungi [19]. Literature data documented an effective control of root-knot nematodes by different Trichoderma species. In greenhouse experiments on tomato infested by *M. javanica*, root galling was reduced and top fresh weight increased following soil pre-treatment with peat-bran preparations of *T. harzianum* [32]. In a recent greenhouse study on the effectiveness of seven indigenous species of Trichoderma for suppressing *M. javanica* infestation on green gram (*Vigna radiata* L.), maximum reductions in the number of nematode galls and eggs were observed for *T. viride* and *T. harzianum* while the lowest suppression was recorded for *T. pseudokoningii* and *T. atroviride* [33]. Analogously, Sonkar et al. [34] documented significant reductions in the number of galls, egg masses, eggs per egg mass and reproductive factors of *M. incognita* on potted tomato following soil application of indigenous isolates of *T. viride*. Moreover, field assessment of the antagonistic potential of seventeen isolates of *Trichoderma* spp. against *M. incognita* on tomato indicated a strong inhibition of nematode reproduction and root galling suppression by *T. asperellum* T-16 and *T. brevicompactum* T-3 [35]. In addition, the in vitro and greenhouse experiments conducted by Zhang et al. [36] proved parasitic and lethal effects of *T. longibrachiatum* and also on the cereal cyst nematode *Heterodera avenae*.

Several mechanisms were documented for the nematicidal activity of Trichoderma species, such as direct parasitism, antibiosis, food competition, induction of plant resistance, and enzymatic hydrolysis [18,24,37,38]. The parasitism of *Meloidogyne* spp. eggs and J2 by *T. harzianum* was attributed to the secretion of several hydrolytic enzymes (chitinases, cellulases, proteases and more) able to degrade the nematode cell wall [39,40]. Analogously, an increased extracellular chitinase activity was reported as the main mechanism of parasitism and inhibition of cysts of *H. avenae* by *T. longibrachiatum* [41]. Singh et al. [42] related the induction of a systemic resistance response in tomato plants treated with *T. harzianum* to an increased production of enzymes, such as phenylalanine ammonia-lyase and peroxidase, known to be involved in systemic resistance. In addition, the data of Martinez Medina et al. [43] indicated that Trichoderma primes jasmonic- and salicylic acid-regulated pathways of resistance to *M. incognita* in tomato roots. Competition for space and nutrients can be also an important mechanism of the phytonematode suppression by Trichoderma species, due to their high and rapid growth capacity [44]. In addition, *Trichoderma* spp. were also reported for producing volatile organic metabolites tested as toxic to different nematode species such as *Panagrellus redivivus*, *Caenorhabditis elegans* and *Bursaphelenchus xylophilus* [45].

Most of treatments with TH included in this study resulted also in a significant enhancement of growth and yield parameters of both tomato crops compared to the non-treated control. However, both tomato yield performance and plant growth were reasonably influenced by seasonal factors, as the higher soil temperatures can have contributed to the larger tomato yield occurred in the second crop yield. Adversely, the higher temperatures in the spring summer crop could have negatively affected the plant growth, favouring the *M. incognita* infestation and its pressure on tomato plants. Plant growth stimulation by *Trichoderma* spp. can be attributed to various mechanisms, such as an enhanced nutrient uptake, a larger solubilization of phosphates and sequestration of inorganic nutrients [24,46]. However, all effects of soil application with Trichoderma-based products, both on nematode infestation and plant growth, are strictly related to the presence of suitable carrier materials. In a recent study, the combination of a polysaccharide-based biopolimer with fungal spores of *T. atroviride* or *T. longibrachiatum* favoured fungal adhesion to tomato roots and nematicidal efficacy of treatments [13]. Moreover, the soilborne phytopathogens *Sclerotinia sclerotiorum* and *Chalara thielavioides* were strongly suppressed by soil treatments with *T. viride* carried on granulated waste fruit pomace [47].

This study also indicated that the combination of TH and OF improved the suppressiveness to *M. incognita* in both tomato crops, while the effects on crop yield were limited only to the first tomato cycle. A previous study of Amir-Ahmadi et al. [48] reported an increased nematicidal activity on *M. javanica* and stimulation of kidney bean plant growth by *T. harzianum* in soils amended with higher amounts of OM. More generally, organic materials, such as composts, were indicated as suitable carrier medium for *T. harzianum*, as stimulating nitrogen mineralization, soil enzyme activity, and fungal growth [49,50].

This study confirmed that the addition of a OM source plays a key role for a successful application of biocontrol agents as Trichoderma. According to their composition, OM sources can directly release nematicidal compounds, increase plant tolerance and resistance and increase soil populations of antagonistic microorganisms [26,27,51]. Moreover, decomposing organic materials represent a rich feeding substrate for bacteriophage nematodes, leading to an increased population of this trophic group, as well as to a reduced space for phytoparasitic species [52,53].

## 4. Materials and Methods

Both autumn and spring tomato cycles were carried out in an unheated greenhouse with a soil of medium texture and a slightly basic reaction (pH 7.2), located at Vitulazio (Caserta, southern Italy). The timetable of all cultural practices, as well as of experimental treatments, is reported in Table 1.

Tomato crop of the previous spring–summer cycle was heavily and uniformly infested by the root-knot nematode *M. incognita*, with a 7.5 average root gall infestation index [54] at the harvest (12 June 2020). The experimental area was uniformly rotavated and then subdivided into 9.6 m^2^ (6 × 1.6 m) plots, aligned along four 1 m spaced parallel rows, according to a randomized complete block design with four replicates per each of the eight treatments.

In the first tomato crop, half of experimental plots were treated with a commercial formulation (Condorsis EC 2020^®^) of the fumigant nematicide 1,3-D, alone or combined with a commercial pellet OF formulation, Stalfert N5^®^ (5% organic N, 30% organic C, 60% OM), as a source of organic matter (OM) and/or a commercial formulate of TH, strain ITEM 908 (Auget^®^). The remaining plots were treated only with OF and TH, either alone or in combination, while four nontreated plots were used as a control (NT). The 1,3-D was applied by drip irrigation at the rate of 180 L ha^−1^, while TH was manually distributed at a 1 kg ha^−1^ rate two days before tomato transplant and again 30 and 60 days after tomato transplant at 0.5 kg ha^−1^. OF (5 and 30% organic nitrogen and carbon, respectively) was distributed on the plot surface at a 2.5 T ha^−1^ dose and then incorporated into the soil one week before transplanting. All treatments barring soil fumigation with 1,3-D were also repeated in the spring tomato crop, at the same rates and in the same plots of the first cycle.

In both crops, four-leaves tomato seedling cv “Shiren F1” were transplanted at a 40 plants/plot density on double rows (80 cm apart into the row and 50 cm in between rows). This hybrid had repeatedly proven to be susceptible to *M. incognita*, either in the same experimental greenhouse or in the farms from the surrounding areas. Presence of phytotoxicity was checked seven days after plant transplanting, substituting failed plants at the same time.

Tomato fruits were harvested four and five times in the first and second crop, respectively. At each harvest, fruit weight was recorded on the ten central plants from each plot. At the final harvest, chlorophyll content (SPAD index), plant top and root weight and Zeck’s root gall infestation index [54] were evaluated on the same ten plants. The values of the SPAD index were determined by five readings on leaves from the central part of the ten sampling plants, by using an atLEAF^®^ CHL PLUS chlorophyll meter combined with atLEAFSoft 1.0 software (FT Green LLC, Wilmington, DE, USA).

In both crops, initial and final soil population density of *M. incognita* was determined on a 10-subsample composite soil sample collected from each plot before the treatments and at harvest, respectively, by extracting nematode juveniles, either already present in soil or emerged from nematode eggs, with the nematode cotton wool filter method [55]. During both crops, the tomato plants received fertilization, irrigation and phytosanitary treatments suggested by the local agronomical technicians.

All data were statistically analysed by the analysis of variance (ANOVA) and means compared by the least significant difference test (*p* ≤ 0.05). All the statistical analyses were performed using the software PlotIT 3.2 (Scientific Programming Enterprises, Haslett, MI, USA).

## 5. Conclusions

The combination of soil fumigation with a source of organic matter and a bionematicide as *T. harzianum* can be an effective model of integrated management of root-knot nematodes. Beyond the well-known biocidal effects of fumigants, the strength of this IPM model also relies on the direct nematicidal activity of the two additional non-chemical components, as well as on their side effects on plant resistance, root system development, nutrient adsorption and interaction among rhizosphere components. As it is not systemic in plants, 1,3-D has low adverse impacts on pollinator insects and has risks to earthworms that are limited to the first growing season after the fumigation treatments [56,57]. Though in the presence of a low environmental risk, biological vacuum effect generated in soil by the fumigation with 1,3-D can be easily reproduced by soil heating treatments (solarization, steam).

As resistance to fumigation treatments can largely vary among different Trichoderma species and strains [28,29], the choice of Trichoderma-based products to include in integrated nematode control strategies should privilege strains that are highly resistant and quickly recolonising, as well as having a strong nematicidal activity. Analogously, attention should also be given to the choice of appropriate sources of organic matter, opting for materials that have already demonstrated a suppressive activity on phytoparasitic nematodes and/or an enhancement of soil antagonistic microbial populations.

## Figures and Tables

**Table 1:** Timetable (month/day/year) of experimental treatments and cultural practices of the two tomato crops.

Operation	1st Crop	2nd Crop
Soil rotavation and plot subdivision	6 July 2020	10 March 2021
Soil sampling	7 July 2020	13 March 2021
Soil fumigation with 1,3-D	17 July 2020	-
Application of the organic fertilizer	13 August 2020	13 March 2021
Application of *T. harzianum*	18 August 2020	18 March 2021
Tomato transplant	20 August 2020	20 March 2021
Application of *T. harzianum*	4 September 2020	19 May 2021
Application of *T. harzianum*	19 September 2020	18 May 2021
Tomato harvest	6 November 2020	7 June 2021
Tomato harvest	20 November 2020	18 June 2021
Tomato harvest	5 December 2020	30 June 2021
Tomato harvest	29 December 2020	10 July 2021
Tomato harvest	-	21 July 2021
Recording SPAD, Zack’ s index, plant weight, soil sampling	29 December 2020	21 July 2021

## Data Availability

All data are available upon request from the corresponding author.
